# Transfusion-Associated Babesiosis after Heart Transplant

**DOI:** 10.3201/eid0901.020149

**Published:** 2003-01

**Authors:** Joseph Z. Lux, Don Weiss, Jeanne V. Linden, Debra Kessler, Barbara L. Herwaldt, Susan J. Wong, Jan Keithly, Phyllis Della-Latta, Brian E. Scully

**Affiliations:** * Columbia University College of Physicians and Surgeons, New York, New York, USA; † New York City Department of Health, New York, New York, USA; ‡ Wadsworth Center, New York State Department of Health, Albany, New York, USA; § New York Blood Center, New York, New York, USA; ¶ Centers for Disease Control and Prevention, Atlanta, Georgia, USA; # Columbia Presbyterian Medical Center, New York, New York, USA

**Keywords:** babesiosis, blood transfusion, heart transplantation, azithromycin, atovaquone, dispatch

## Abstract

We describe a 54-year-old spleen-intact man with transfusion-associated *Babesia microti* infection after a heart transplant. Adult respiratory distress syndrome developed in the patient, and he required mechanical ventilation. Our experiences with this patient suggest that babesiosis should be considered in the differential diagnosis of transplant patients who have fever and hemolytic anemia.

Babesiosis is a tick-borne protozoan illness caused by infection of erythrocytes with various species in the genus *Babesia*. In the United States, *Babesia microti* is the agent most commonly reported to cause human infection (*1*). More recently, the MO1-type, WA1-type, and CA1-type *Babesia* species have been identified as causing clinical disease in the United States (*2*–*5*). *Babesia* infection can also be acquired by blood transfusion (*2*,*6*,*7*). More than 40 cases of transfusion-transmitted *B. microti* infection have been reported in the United States (R. Cable and B. Herwaldt, unpub. data). *B. microti* infection is often asymptomatic (*8*) but may cause a malaria-like illness characterized by fever and hemolytic anemia. Babesiosis can also be associated with severe complications that include renal failure (*9*,*10*), disseminated intravascular coagulation (*9*), and adult respiratory distress syndrome (*1*,*9*,*10*). The risk of developing this clinical infection is increased for elderly, asplenic, or immunosuppressed patients (*11*). Here we describe a case of *B. microti* infection in a 54-year-old spleen-intact man acquired by blood transfusion after cardiac transplantation.

## Case Report of Blood Recipient

The patient, a 54-year-old resident of New Jersey, had a medical history of coronary artery disease and atrial fibrillation. Approximately 18 months before his transplant, a “bull’s-eye” rash developed, and the patient was empirically treated for Lyme disease. He did not recall a tick bite.

On August 19, 2000, he had an anterior wall myocardial infarction and was hospitalized in his hometown. He required intubation, placement of a coronary stent and an intra-aortic balloon pump, use of intravenous cardiac inotropes, and transfusion of 2 U of packed red blood cells (PRBC). When fever developed, he was empirically treated with vancomycin, ciprofloxacin, and metronidazole without improvement. No source of infection was found. On September 6, he was transferred to a New York City medical facility for placement of a left ventricular assist device. *Staphylococcus epidermidis* was isolated from two sets of blood cultures*.* He was then treated with vancomycin and trimethoprim-sulfamethoxazole for 4 weeks.

On September 26, the patient received an orthotopic cardiac transplant. He received 32 U of irradiated leukocyte-reduced PRBC, 23 U of fresh frozen plasma (FFP), 6 U of irradiated platelets, and 4 U of cryoprecipitate during his 5.5-week hospital stay. On October 16, he was discharged on an immunosuppression regimen of cyclosporine, mycophenolate, and prednisone.

On November 5, the patient became febrile. He was evaluated by his cardiologist 3 days later. He had fever, chills, diaphoresis, headache, and sore throat. Blood and throat cultures were obtained, as well as an endomyocardial biopsy specimen. No source of infection or evidence of cardiac rejection was found.

On November 9, he was hospitalized again for evaluation of continued fever. On admission, his temperature was 38.4°C. His surgical wound had healed well. Laboratory tests showed a leukocyte count of 4.3 x 10^3^/mm^3^ (83% neutrophils, 9% lymphocytes, 8% monocytes), hemoglobin concentration of 11.4 g/dL, and a platelet count of 54,000/mm^3^. On November 10, the staff of the hospital parasitology laboratory identified intraerythrocytic ring forms and tetrads consistent with *B. microti* infection on a peripheral blood smear; these results were confirmed by the Centers for Disease Control and Prevention (CDC). The parasitemia level was 1.6%. Indirect fluorescent antibody (IFA) testing (*12*) of his serum at CDC showed that his IFA titers had risen from <1:8 (i.e., negative) pretransplant to 1:1024 posttransplant (Table). Nested polymerase chain reaction (PCR) analysis of blood, using primers described previously (*13*), was positive for *B. microti* DNA. Results of pre- and posttransplant serologic testing for antibodies to *Borrelia burgdorferi*, *Ehrlichia chaffeensis*, and the agent of human granulocytic ehrlichiosis (*14*), performed at the New York State Department of Health, were negative (Table).

**Table:** Results of serologic testing, polymerase chain reaction analyses, and hamster injection for specimens from the case-patient with babesiosis and the implicated blood donor^a^

Date of specimen	Timing of specimen	Test	Result of testing
**Case-patient**			
September 21, 2000	Pretransplant	*Babesia microti* IFA	<1:8^b^
November 14, 2000	Posttransplant	*B. microti* IFA	1:1024
November 14, 2000	Posttransplant	*B. microti* PCR	Positive
September 21, 2000	Pretransplant	Agent of HGE, IgM^c^	Negative
November 14, 000	Posttransplant	Agent of HGE, IgM	Negative
September 21, 2000	Pretransplant	Agent of HGE, IgG	Negative
November 14, 2000	Posttransplant	Agent of HGE, IgG	Negative
September 21, 2000	Pretransplant	*Ehrlichia chaffeensis* IFA	Negative
November 14, 2000	Posttransplant	*E. chaffeensis* IFA	Negative
September 21, 2000	Pretransplant	Lyme ELFA	Negative
November 14,2000	Posttransplant	Lyme ELFA	Negative
**Implicated blood donor (tested approximately 5 months after donation)**			
February 28, 2001	Postdonation	*Babesia microti* IFA	1:256
February 28, 2001	Postdonation	*B. microti* PCR	Negative
February 28, 2001	Postdonation	Hamster inoculation^d^	Negative

^a^IFA, indirect fluorescent antibody; PCR, polymerase chain reaction; HGE, human granulocytic ehrlichiosis; Ig, immunoglobulin; ELFA, enzyme-linked fluorescence assay.  **^b^*Babesia microti* IFA≤ 1:8=negative. ^c^IgM and IgG immunoblots for HGE.** ^d^Two hamsters were each inoculated intraperitoneally on April 3 with 0.75 mL of blood obtained on February 28. Giemsa-stained smears were made from hamster blood obtained twice weekly, for 8 weeks, by tail snip. The hamsters did not become demonstrably parasitemic

On November 10, therapy with quinine (650 mg orally, 3 times a day) and clindamycin (400 mg intravenously, 4 times a day) was begun. His course was complicated by worsening parasitemia (maximum documented level was 3.1% on November 13), hemolytic anemia (hemoglobin concentration decreased from 11.4 g/dL on November 9 to a minimum value of 7.4 g/dL on November 15), acute renal failure (creatinine value rose from 1.4 mg/dL on November 9 to a maximum level of 3.3 mg/dL on November 15), and adult respiratory distress syndrome requiring mechanical ventilation.

On November 14, his therapy for babesiosis was changed to azithromycin (500 mg intravenously, once a day) and atovaquone (750 mg orally, 2 times a day) because of severe tinnitus, increased level of parasitemia (3.1% on November 13), and ongoing fever (maximum of 38.6°C). On November 21, he was afebrile and had a negative blood smear. He was discharged on November 25. Therapy with azithromycin (250 mg orally, once a day) and atovaquone (750 mg orally, 2 times a day) was continued for a total of 2 weeks on these drugs. The patient remained well as of August 2001.

## Investigation of Blood Donors

We traced the donors of the 32 PRBC units transfused during the patient’s hospitalization in New York City. For two donors, the transfused units had associated frozen components (i.e., FFP) available that could be retrieved; for six donors, PRBC units from subsequent donations were available for testing. The blood products from these eight donors were negative for antibodies to *B. microti* by IFA testing.

The other 24 donors submitted blood for testing. On March 12, 2001, the New York State Department of Health laboratory reported that one donor had an IFA titer of 1:256, which was confirmed by CDC (Table). The other 23 donors had negative IFA titers. The blood specimen from the implicated donor had been obtained on February 28, 2001, 5 months after the original blood donation on September 22, 2000. The PRBC unit was transfused on October 1, 2000, and the patient first reported fever on November 5, 2000. Thus, the incubation period for the case of babesiosis was estimated to be 35 days. CDC performed additional diagnostic testing on the implicated donor’s blood. PCR analysis was negative for *B. microti* DNA, and parasitemia did not develop in hamsters injected with his blood (Table). The donor was thus implicated on the basis of serologic rather than parasitologic data. Given that he was not treated for babesiosis, the negative results of the parasitologic testing suggest that the donor’s infection cleared spontaneously. However, he could have had low-level or intermittent parasitemia not detected by the parasitologic testing.

After the donor was implicated, other components from the index donation were investigated. No other patients received blood components from the implicated donation. The only other component identified was one unused unit of FFP, which already had been destroyed. The donor’s only previous donation was 1 year before the index donation. The lone recipient from that donation remained asymptomatic but was not tested for evidence of *B. microti* infection.

## Investigation of Implicated Donor

The implicated donor, a 45-year-old spleen-intact man from Westchester County, New York, had been asymptomatic the year before his blood donation. In 2000, he had visited New Haven County, Connecticut, in May; Long Island (Jones Beach), New York, in July and August; and Narragansett, Rhode Island, in July. He worked outdoors and gardened as a hobby. He did not recall a tick bite.

## Investigation of Heart Donor

The heart donor was a 54-year-old woman who died after a cerebrovascular accident. She had lived and worked in New York City and had had no symptoms consistent with babesiosis before the stroke. While hospitalized, she had remained afebrile with a normal complete blood count and renal function. No serum from her was available for *B. microti* IFA testing.

## Conclusions

We conclude that our patient, who lived in New Jersey, acquired babesiosis through transfusion of PRBC in New York City after a cardiac transplant. The evidence consistent with this conclusion includes the following: he did not recall a tick bite (most patients with babesiosis do not [*9–11*]); he did not spend much time outdoors; he was seronegative by *B. microti* IFA 5 days before his heart transplant and markedly seropositive when babesiosis was diagnosed 45 days after his transplant; he received blood from a donor with a *B. microti* IFA titer of 1:256; and he became symptomatic during the typical two 8-week incubation periods for transfusion-transmitted *B. microti* infection (*7*).

Other possible modes of transmission seem much less likely. We doubt he became infected through a blood transfusion in New Jersey; he was seronegative by *B. microti* IFA 4.5 weeks after the New Jersey transfusions. We also doubt that he acquired his infection while recuperating after his heart transplant; his limited outdoor activities made tick exposure unlikely.

More than 40 cases of transfusion-transmitted babesiosis have been reported in the United States. Most infections have been acquired through PRBC transfusions, but cases have also resulted from transfusion of frozen-deglycerolized red cell units (*15*,*16*) and platelet units (*6*,*7*) (the latter have residual red cells). Two previous cases of *B. microti* infection have been reported in solid-organ transplant patients, although no cases acquired through transplantation per se have been reported (*17*,*18*).

We initially treated our patient with quinine and clindamycin but later changed the regimen to atovaquone and azithromycin. A recent clinical trial demonstrated that the combination of atovaquone and azithromycin is as effective as quinine and clindamycin for the treatment of babesiosis in patients without life-threatening disease (such patients were excluded from this trial) (*19*). In our patient, who was critically ill, immunosuppressed, and a solid-organ transplant recipient, the combination of atovaquone and azithromycin was effective therapy.

Babesiosis should be considered in the differential diagnosis of transplant patients who have fever and hemolytic anemia, and blood transfusion should be considered as one of the possible means of acquisition of infection. Solid-organ transplant recipients may receive many blood products, which necessitates an extensive investigation to implicate a donor.
