# Correction, Vol. 9, No. 1

**DOI:** 10.3201/eid0902.020149

**Published:** 2003-02

In “Transfusion-Associated Babesiosis after Heart Transplant” by Joseph Z. Lux et al., errors occurred in the text. On page 118, left column, lines 20–22, the sentence should read “he became symptomatic during the typical 2- to 8-week incubation period for transfusion-transmitted *B. microti* infection (*7*).”

The corrected text appears online at http://www.cdc.gov/ncidod/eid/vol8no12/02-0149.htm.

